# Some Observations on the Treatment of the Puerperal Fever

**Published:** 1821-02

**Authors:** Edward Jukes

**Affiliations:** Surgeon.


### *Some Observations on the Treatment of the Puerperal Fever.*

By Mr. Edward Jukes, Surgeon.

To produce observations on the treatment of the puerperal fever (*puerperal peritonitis*), after those of Ramsbotham, Gordon, Hey, and, especially, the treatise of Dr. Armstrong, —in which the nature of the disease, the indications for its cure, and the rules for the application of the remedies, are established in such a way as to place them above either doubt or disputation,—may appear to be both unnecessary and unprofitable. The anticipation of such considerations would have induced me to refrain from taking up the pen to write on this subject, had I not had frequent occasions for remarking that the existing information comprised in books, is not that of a considerable proportion of the class of medical practitioners on which the welfare of patients, in this disease, must chiefly depend; for it is to them,—that is to say, the surgeon-apothecaries,—that the management of the generality of patients is confided during the stage of the disease in which, for the most part, medical aid is alone successfully employed. A great proportion of the class of practitioners just named, consists of men either too much occupied by actual practice to be able to read much, if they are disposed to it; or who have not leisure or ability for that degree of reflection which is requisite in order that the inferences stated in, or drawn from, books, may be applied to particular cases with appropriate energy and precision. I am aware that it may be asked here,—why I add, then, to the number of writings which, according to my acknowledgment, must be devoid of utility? My reply is, that the intercourse I have had with practitioners of my own class has permitted me to perceive the deficiencies of some of them, and the means of supplying those deficiencies, better, perhaps, than our esteemed authors, who are apt to suppose that all medical men can reason analytically and inductively as well as themselves. There are practitioners who have understood that it has been stated that blood-letting and purgatives are the only means to be depended on for the cure of the puerperal fever, and that, without them, all other measures will fail of success. They have, accordingly, employed blood-letting and purgatives, and, their patients having died, they have ceased to regard the practice in a favourable manner. They have heard of cures apparently effected by large doses of opium or other powerful stimulants, and, having witnessed fortunate results from their use in late and desperate stages of the disease, they have employed them in all stages of it; and, of course, with consequences that make them regard the puerperal fever with sentiments of hopeless anxiety on all occasions.

Such results as these ensue from the want of discrimination in marking the stages of the disease to which the several remedies are exclusively or especially appropriate, as well as of sufficient precision in the application of them to particular cases. I have, of late, treated many cases of the puerperal fever, and with nearly universal success; yet I do not pretend to have discovered a single important fact respecting the nature of the disease, or to have acted in conformity with any one precept which I have not derived from the authors above enumerated. I have merely collected their conclusions; generalized them to a certain extent for my own use, and made the principles thus formed the general guides for my conduct, to be deviated from in some degree as particular circumstances might indicate. A person who would draw up a series of aphorisms, embracing the general indications and precepts for the treatment of the puerperal fever, would, I think, confer a great benefit on many members of the profession. It is much to be regretted that this has not been effected by our latest and justly much esteemed writer on the subject. There is, I know, this objection to general principles for the treatment of disease, that they must be either too vague or too exclusive to be applied in a precise manner to particular cases; and, therefore, that, if acted on, they must occasionally be the cause of error. In opposition to this it may be stated, that some general principles of conduct are absolutely necessary: no man can act without them; and it is much easier to discern exceptions, than it is to arrive at them by inductive reasoning; which, when it rests on acknowledged data, is the pillar of true science, the basis of which is fact, whilst truth is its capital: but, inductive reasoning, or theory, has been too often confounded with hypothesis, which sets out in search of data, instead of proceeding from established facts; and, though hypothesis has often proved the wings of science, and has even directed its course, in a flight bold but eccentric, like that of the eagle, towards the source of light, it has more frequently led to wanderings which may be compared with those of a person who, on rising from the perusal of his Plato, should embark for the fabled Atlantides.

The puerperal fever is sufficiently distinct and characterized a morbid state, and experience has sufficiently well established the principles for its treatment, to admit of the general inferences above alluded to. It is well ascertained that, whether or not it essentially depends on peritoneal inflammation, the danger is always in a direct ratio with the intensity of such inflammation; and that there is no form of inflammation, the different degrees and consequences of which succeed each other with more rapidity, or in which the period of irritation has a more short duration, especially when it tends to a fatal issue. Some pathologists, apparently overlooking the first stage of the disease, the shivering with which it is ushered in, the acute and constant pains, with excessive tenderness, in the abdomen; the burning heat and aridity of the skin; and the frequent, full, and hard pulse with which it is accompanied; and, considering alone the state of collapse, debility, cold sweats, rapid and feeble pulse, and signs of great exhaustion especially of nervous power, have viewed in the disease only a state of debility, and have not considered that this debility is merely a consequence of the previous excitement, and that the extent of the former is in a direct ratio to the intensity and duration of the latter. There are men of long experience, I am aware, who think differently of the nature of the disease, chiefly because they have found it terminating favourably, in some cases, under the use of cordials and stimulants; and perhaps they have forgotten the remark of Sydenham respecting the treatment of the small-pox, that there are cases which will not prove fatal, in spite of bad management; or they cite authorities from old writers, and the zeal of some of them for such precepts has led them, when the inflammatory nature of the disease has been demonstrated by dissection after death, to imitate the conduct of Silvius, the preceptor of Vesalius, who, when confounded by the demonstrative proofs of his pupil, rather than doubt the infallibility of Galen, asserted that "men were otherwise made in the days of Galen than in the time of Vesalius."

The foregoing views of the nature of the disease, with a consideration of the peculiarly irritable state of the nervous system, and of the violent derangements of function dependent on it, furnish the first indications for the treatment; and, in conformity with them, experience has shown that blood-letting, employed in the first stage of the inflammation, (generally within thirty-six hours from its commencement; though Dr. Armstrong relates some cases in which it was resorted to at a later period with favourable results,) and copiously, as well as promptly, is absolutely necessary in almost every case, in order to lead it to a favourable issue. After the first stage of irritation has passed away, and real debility and collapse have come on, (and, when the disease has proceeded unalleviated, these generally occur in from twenty or thirty hours to two or three days,) blood-letting, by venesection, is then always injurious; and the application of leeches to the abdomen, is not often, though it is sometimes, advisable. In a great many cases, blood-letting, to the extent of from fifteen to five-and-twenty ounces, effected within a very few hours from the onset of the disease, has arrested it almost instantly; and but a small proportion of cases treated thus, (with the other means to be presently mentioned,) with a repetition of the measure, in some instances, after a few hours, have terminated fatally; excepting only instances where the disease has occurred epidemically, and in Lying-in Hospitals, and when it has been accompanied with habitual or recently-acquired considerable derangement and debility of the nervous system; or when it has been accidentally accompanied with other diseases.

Next in importance to blood-letting, and a measure which should always accompany it when diarrhœa does not spontaneously occur, is the use of purgatives: and experience has shown that a medicine of this kind which will act promptly and affect the whole intestinal tube, such as a scruple of calomel, with as much jalap, or salts and senna in sufficient quantities, administered in the commencement of the disease, and followed by the constant and regular exhibition of milder purgatives, such as castor-oil, or sulphate of magnesia and senna, manna, &c. so as to keep up a gentle diarrhœa for several days, have led many severe cases to a favourable termination. But hardly any case can, perhaps, occur, in which it would be justifiable to trust to purgatives alone. These remedies should accompany the use of blood-letting, and be employed with the same promptness as the latter measure. There may be cases where there has been great exhaustion from previous hemorrhage, or some long-continued or very severe disease, or some extraordinary derangement of the nervous system, in which general blood-letting may not be advisable; but none in which leeches, to the number of twenty or thirty, should not be applied to the abdomen, if venesection is not employed, and often when it is practised. The puerperal fever is generally accompanied with, and often has been preceded by, a costive state of the bowels; but it should not hence, or because purgatives act beneficially, be considered that the costiveness has produced the disease. Common peritonitis, from whatever cause, is ordinarily attended, in its first stage, with constipation. The irritation of the peritoneal coat of the intestines seems to suspend or lessen their peristaltic action, and to diminish the secretions from their mucous membrane; and producing irritation of the latter, by purgatives, relieves that of the peritoneum, probably, on the principle of revulsion; or just for the same reason that a patient, whose case is related in the first volume of the Dublin Hospital Reports, by Dr. Cheyne, had diarrhœa and ascites repeatedly alternating with each other. Sometimes the puerperal fever is accompanied with a spontaneous diarrhœa; and, when this happens in a violent degree at the commencement, it ordinarily marks an unusually severe form of the disease, which is not unfrequently fatal. In one case of this kind, in which I had an opportunity of examining the body after death, I found the mucous membrane of nearly the whole extent of the intestines had been in a state of inflammation, in addition to that affection of the whole of the peritoneum. In other cases, as far as my own observations have extended, the mucous tunic has been apparently in the most healthy state; whilst the peritoneum has been red, thickened, coated with false membranes, or gangrenous in patches, and the abdomen filled with serous and purulent fluids.

It must be obvious that when diarrhœa of the kind just mentioned takes place, irritating purgatives should not be employed; and the administration of astringents or cordial opiates, so often used, is equally injurious. Mild mucilaginous drinks and emollient enemas should be given, with ipecacuanha in small and frequently repeated doses, with or without kermes mineral, after the manner of Doulcet. Ipecacuanha is, in most cases, beneficial after the first stage of the disease has passed, and it may be combined with the gentle purgatives which may be employed. Very obstinate constipation is an unfavourable sign; and, indeed, a very great proportion of the fatal cases on record, seem to have been accompanied, in the first stage, with either this symptom or severe diarrhœa.

But, in pursuing these remarks, I might appear to be attempting to effect what it is really my intention to point out for the attention of others, who are better qualified for the task: I shall therefore conclude this desultory paper with the history of a case of well-marked puerperal fever; which I adduce, because it exemplifies in a very forcible manner the powers of copious blood-letting as a remedy for that disease.

Mrs. Martha Clark, aged 24, of a healthy and rather robust constitution, residing at No. 40, York-street, Westminster, was delivered of her first child about nine o'clock in the evening of the 7th of September, 1820, after a labour regular in its progress, short in its duration, and favourable in its termination.

*Sept. 8th.*—She has passed a good night; is remarkably cheerful, and has been free from pain; the lochiæ have appeared in the ordinary way; the pulse 80; the skin moist. No motion since delivery.

*9th; eight o'clock in the morning.*—Violent pain in the abdomen (principally between the umbilicus and the pubis) came on late last night, occurring every ten or twelve minutes, and continuing between two and three minutes. Within the last few hours the pain has been constant; has ceased to be accompanied with a sense of bearing-down, as it was at first, and the slightest pressure increases it considerably; the weight of the hand is intolerable, and the increase of pain by pressure does not subside by a continuation of the pressure, as is commonly the case in *after-pains.* Pulse 130; skin hot and dry; tongue white; bowels confined, and the lochiæ suppressed. Eighteen ounces of blood were drawn, and one ounce of castor-oil given; fomentations ordered to the abdomen.

11 *o'clock, a. m.*—Pain continues with increased violence. Eighteen leeches applied to the abdomen, and the hot fomentations continued; a scruple of calomel was given, and a mixture of salts and senna directed to be taken every three hours.

4 *o'clock, p. m.*—The pains not diminished, and the tenderness of the abdomen so extreme that the weight of the bed-clothes is insupportable; pulse still 130. One motion obtained. The purgative mixture to be continued.

9, *p.m.*—The pain is still more severe, is extended equally over the whole of the abdomen, the tenderness of which is so exquisite, that, on a small piece of dry sponge falling on it, outside the bed-clothes, from the height of about two feet, the patient screamed out in an accent of extreme suffering. She lies on her back, with her arms bent, and the thighs extended apart and a little raised, so as to prevent the weight of the bed-clothes from falling on the belly. There is an expression of much anguish in the countenance, and the face is bedewed with a greasy-looking sweat. The skin generally is cold and damp. Inspiration is short, and evidently restrained by the efforts of the patient. The tongue is covered with a thick white fur. The pulse is 140, hard, small, and thrilling. The bowels have been open once since the last report. The lochiæ are suppressed. The patient sighs, complains, in an interrupted voice, of languor and faintness, and says she is dying. The blood drawn in the morning is cupped and a little buffy.

Twenty ounces of blood were taken from the arm. Immediately after the bleeding the pulse had fallen to 90, and was softer and fuller; the breathing was much deeper, and apparently unrestrained by voluntary exertions.

A blister was applied to the interior part of each thigh; an enema of gruel, sulphate of magnesia, and oil, administered; and a draught of one grain of ipecacuanha, with two drachms of sulphate of magnesia, in infusion of roses, given every four hours.

*10th*, 8, *a.m.*—The pain subsided soon after the bleeding last evening; and the patient fell asleep about midnight, and slept for several hours. She is now almost wholly free from pain, and the tenderness of the abdomen is but very slight. The pulse is 76, and in all respects has a healthy character. The anxiety in the countenance has disappeared, and the face is more florid; the skin generally is moist and warm. The bowels have been opened several times; there is nothing remarkable in the appearance of the stools. The draughts to be continued.

*11th.*—Has had no return of any of the symptoms of the disease, and is convalescent. The lochiæ have re-appeared. The draughts to be continued after longer intervals, so as to keep the bowels in a relaxed state.

*15th.*—Is perfectly well, and has suckled the child for the last two days.

*Nov. 1st*, 1820.

